# Combination Therapy with UV-4B and Molnupiravir Enhances SARS-CoV-2 Suppression

**DOI:** 10.3390/v15051175

**Published:** 2023-05-16

**Authors:** Evelyn J. Franco, George L. Drusano, Kaley C. Hanrahan, Kelly L. Warfield, Ashley N. Brown

**Affiliations:** 1 Institute for Therapeutic Innovation, Department of Medicine, College of Medicine, University of Florida, Orlando, FL 32827, USA; e.franco@ufl.edu (E.J.F.); george.drusano@medicine.ufl.edu (G.L.D.); kaley.hanrahan@medicine.ufl.edu (K.C.H.); 2 Department of Pharmaceutics, College of Pharmacy, University of Florida, Orlando, FL 32827, USA; 3 Emergent BioSolutions, Gaithersburg, MD 20879, USA; warfieldk@ebsi.com

**Keywords:** UV-4B, EIDD-1931, combination therapy, SARS-CoV-2, COVID-19, variant

## Abstract

The host targeting antiviral, UV-4B, and the RNA polymerase inhibitor, molnupiravir, are two orally available, broad-spectrum antivirals that have demonstrated potent activity against SARS-CoV-2 as monotherapy. In this work, we evaluated the effectiveness of UV-4B and EIDD-1931 (molnupiravir’s main circulating metabolite) combination regimens against the SARS-CoV-2 beta, delta, and omicron BA.2 variants in a human lung cell line. Infected ACE2 transfected A549 (ACE2-A549) cells were treated with UV-4B and EIDD-1931 both as monotherapy and in combination. Viral supernatant was sampled on day three when viral titers peaked in the no-treatment control arm, and levels of infectious virus were measured by plaque assay. The drug–drug effect interaction between UV-4B and EIDD-1931 was also defined using the Greco Universal Response Surface Approach (URSA) model. Antiviral evaluations demonstrated that treatment with UV-4B plus EIDD-1931 enhanced antiviral activity against all three variants relative to monotherapy. These results were in accordance with those obtained from the Greco model, as these identified the interaction between UV-4B and EIDD-1931 as additive against the beta and omicron variants and synergistic against the delta variant. Our findings highlight the anti-SARS-CoV-2 potential of UV-4B and EIDD-1931 combination regimens, and present combination therapy as a promising therapeutic strategy against SARS-CoV-2.

## 1. Introduction

Severe Acute Respiratory Syndrome Coronavirus 2 (SARS-CoV-2), the cause of COVID-19, is a respiratory virus belonging to the family *Coronaviridae* [1]. Following its emergence in late 2019 [2], SARS-CoV-2 quickly became a substantial threat to global public health due to its high transmissibility and the risk of severe complications associated with infection. The severity of acute COVID-19 infection varies, with most patients experiencing mild/moderate symptoms; however, severe cases are characterized by life-threatening symptoms, including acute respiratory distress syndrome (ARDS), respiratory failure, sepsis, and multi-organ failure [1,2]. In addition, COVID-19’s detrimental effect on patient quality of life can also extend beyond acute infection [3] since symptoms such as fatigue, dyspnea, and loss of taste or smell may persist for weeks to months [3,4].

In an effort to curb viral transmission, a number of vaccines have been developed and approved worldwide [5,6]. Vaccination has proven effective in preventing or reducing the severity of infection; however, breakthrough cases of infection can occur in fully vaccinated patients [7]. In addition, concerns over the effectiveness of vaccines have surfaced following the emergence of new variant strains [5,7]. The limitations associated with vaccination highlight the need for effective antiviral therapies against SARS-CoV-2.

For these studies, we selected two orally available antivirals, UV-4B and EIDD-1931, which have demonstrated antiviral activity against numerous RNA viruses [8,9,10,11,12,13]. UV-4B is a host targeting agent that has been investigated for its antiviral potential against influenza viruses, dengue virus and SARS-CoV-2 [8,9,10,14]. UV-4B interferes with the proper folding of viral glycoproteins by inhibiting the endoplasmic reticulum enzymes α-glucosidase I and II [10,15]. Because enveloped viruses rely on the host cell glycosylation machinery for the proper processing of viral glycoproteins, inhibition of these enzymes can hinder viral assembly/release and/or reduce viral infectivity [15]. Molnupiravir (MOL; EIDD-2801) is an RNA polymerase inhibitor that has been investigated for activity against several RNA viruses, including influenza viruses, SARS-CoV-2, Middle East respiratory syndrome coronavirus (MERS-CoV), and SARS-CoV, among others [13]. MOL is an isopropyl ester prodrug of β-D-N4-hydroxycytidine (NHC; EIDD-1931), a nucleoside analogue that inhibits viral replication through activity as a mimic of cytidine or uridine [16]. Upon administration, plasma esterases rapidly convert MOL into EIDD-1931, which is then taken up by the host cell and converted into an active triphosphate metabolite, EIDD-1931-TP, by host cell kinases [17]. The incorporation of EIDD-1931-TP into nascent RNA strands is thought to curb viral replication due to an accumulation of mutations in the viral genome [18]. Because EIDD-1931 is the major metabolite circulating in plasma, antiviral evaluations in this work will be conducted using EIDD-1931 rather than the prodrug MOL. The goal of this work is to evaluate the antiviral potential of combination regimens of UV-4B and EIDD-1931 against the SARS-CoV-2 beta, delta, and omicron BA.2 variants in ACE2-A549 cells, a human lung cell line.

## 2. Materials and Methods

### 2.1. Cells, Virus and Antivirals

A549 cells that express the angiotensin-converting enzyme 2 (ACE2) receptor (ACE2-A549 cells) were generously provided by Dr. Shinji Makino [19]. ACE2-A549 cells were cultured in Dulbecco’s modified Eagle’s medium (DMEM) (Hyclone, Logan, UT, USA) and supplemented with 10% fetal bovine serum (FBS) (Sigma Aldrich; St. Louis, MO, USA) and 1% penicillin-streptomycin solution (Hyclone, Logan, UT, USA). Vero E6 cells (ATCC CRL-1586) were purchased from the American Type Culture Collection (ATCC, Manassas, VA, USA), maintained in minimum essential medium (MEM) (Corning Cellgro, Manassas, VA, USA), and supplemented with 10% FBS (Sigma Aldrich, St. Louis, MO, USA) and 1% penicillin-streptomycin solution (Hyclone, Logan, UT, USA). Vero E6 cells that express the human transmembrane serine protease 2 (TMPRSS2) enzyme and human angiotensin-converting enzyme 2 (ACE2) (Vero E6-TMPRSS2-T2A-ACE2) were obtained from Biodefence and Emerging Infectious Resources Repository (BEI Resources; Manassas, VA, USA), cultured in DMEM (Hyclone, Logan, UT, USA), and supplemented with 10% FBS (Sigma Aldrich, St. Louis, MO, USA) and 10 μg/mL puromycin (Thermo Fisher Scientific; Waltham, MA, USA). Cells were incubated at 37 °C, 5% CO_2_, and subcultured twice weekly.

The beta variant of SARS-CoV-2 (isolate hCoV-19/South Africa/KRISP-EC-K005321/2020) was acquired from Biodefense and Emerging Infectious Resources Repository (BEI Resources; Manassas, VA, USA). The delta variant of SARS-CoV-2 (lineage B.1.617.2, strain designation GNL-1205) and the omicron variant (lineage BA.2, strain designation MDL-1217) were acquired from the UTMB Arbovirus Reference Collection (Galveston, TX, USA). Beta and delta stock viruses were propagated on Vero E6 cells, and the omicron BA.2 variant was propagated on Vero E6-TMPRSS2-T2A-ACE2 cells. Viral titers were determined by plaque assay on either Vero E6 (beta and delta variants) or Vero E6-TMPRSS2-T2A-ACE2 (omicron variant) cells as previously described [20].

UV-4B was a generous gift from Emergent BioSolutions (Gaithersburg, MD, USA). Stock solutions of 2809.8 μM (1000 μg/mL) were prepared in sterile deionized water. EIDD-1931 was obtained from MedKoo Biosciences (Morrisville, NC, USA). EIDD-1931 stocks of 1000 μg/mL were reconstituted in 100% dimethyl sulfoxide (DMSO).

### 2.2. Antiviral Evaluations

ACE2-A549 cells were seeded into 6-well plates and incubated overnight at 37 °C, 5% CO_2_. The following day, cells were infected with either the beta, delta, or omicron variants of SARS-CoV-2 at multiplicities of infection (MOIs) of 0.0075, 0.01, or 0.05, respectively. Plates were incubated for one hour, the viral inoculum was removed, and the unbound virus was washed away following two wash steps with warm PBS. For monotherapy evaluations, drug concentrations ranging from 0 to 142.36 μg/mL UV-4B and 0 to 5 μg/mL EIDD-1931 were evaluated. Viral supernatants were sampled daily over 3 days, clarified by high-speed centrifugation, and stored at −80 °C until the end of the study. Viral burden was quantified by plaque assay on Vero E6 cells for the beta and delta variants, and Vero E6-TMPRSS2-T2A-ACE2 cells for the omicron variant.

For combination studies, ACE2-A549 cells were seeded into 6-well plates and infected as described above, and drug concentrations ranging from 0 to 9 μg/mL for UV-4B and 0 to 0.5 μg/mL for EIDD-1931 were evaluated either alone or in combination against the beta and omicron variant strains of SARS-CoV-2. In addition, UV-4B concentrations ranging from 0 to 27 μg/mL and EIDD-1931 concentrations ranging from 0 to 1 μg/mL were employed for evaluations of the antiviral effect against the delta variant strain of SARS-CoV-2. Plates were maintained at 37 °C, 5% CO_2_. Viral supernatant samples were harvested on day 3, as this time point corresponded to the day when peak levels of infectious virus were attained in the no-treatment control arm of each study. Samples were clarified by high-speed centrifugation and then stored at −80 °C. Infectious titers were measured by plaque assay on Vero E6 or Vero E6-TMPRSS2-T2A-ACE2 cells as previously described [9]. Of note, the plaque assay procedure for studies conducted with the omicron and delta variants was slightly modified such that samples were assayed undiluted in order to lower the assay limit of detection and increase the dynamic range of the virus that could be quantified.

### 2.3. Statistical Analysis

EC_50_ values from monotherapy studies were determined by calculating the area under the viral burden time curve (AUC_VB_) for each regimen over the course of the experiment. AUC_VB_ values were plotted against the corresponding drug concentration, and an inhibitory sigmoid E_max_ model was fitted to the data using GraphPad Prism version 7.02 (GraphPad Software; La Jolla, CA, USA).

### 2.4. Mathematical Modeling

The Greco Universal Response Surface Approach (URSA) model was used to assess the interaction for antiviral effect between UV-4B and EIDD-1931 [21,22,23,24]. The equation for the model is:

(1) $$1=\frac{D_{1}}{EC_{50,\;1\;}{\left(\frac{E}{E_{con}-E}\right)}^{1/m_{1}}}+\frac{D_{2}}{EC_{50,2\;}{\left(\frac{E}{E_{con}-E}\right)}^{1/m_{2}}}\;\;\;\;\;\;\;\;+\frac{\alpha D_{1}D_{2}}{EC_{50,1\;}EC_{50,2\;}{\left(\frac{E}{E_{con}-E}\right)}^{(1/2m_{1}+1/2m_{2)}}}$$

where E corresponds to viral replication; D_1_ is the concentration of UV-4B; D_2_ is the concentration of EIDD-1931; EC_50,1_ is the UV-4B concentration that causes half maximal antiviral effect; EC_50,2_ corresponds to the concentration of EIDD-1931 that causes half maximal effect; m_1_ and m_2_ are Hill’s constants for UV-4B (m_1_) and EIDD-1931 (m_2_); E_con_ corresponds to viral replication in the absence of drug; and lastly, α is the interaction parameter. In the event that α and its 95% confidence interval (CI) cross 0, the interaction is considered additive. The effect is synergistic if α is >0 and its 95% CI do not cross 0. An effect is deemed antagonistic if α < 0 and its 95% CI do not cross 0. Data were analyzed in the ID module of the ADAPT5 software package [25].

### 2.5. Cytotoxicity Evaluations

The UV-4B and EIDD-1931 monotherapy and combination regimens assayed in our antiviral evaluations were also investigated for their effect on cell health using the WST-1 cell proliferation assay (Roche Diagnostics GmbH; Mannheim, Germany) as previously described [9]. The effect of each regimen on cell viability was determined by calculating percent cell viability relative to untreated cells.

## 3. Results

### 3.1. UV-4B and EIDD-1931 Monotherapy Regimens Inhibit Replication of Multiple SARS-CoV-2 Variants

We have previously shown that single-agent therapy with UV-4B effectively inhibited replication of the beta variant of SARS-CoV-2 in ACE2-A549 cells [9]; UV-4B exhibited an EC_50_ value equivalent to 0.876 μg/mL against this variant following three days of drug exposure (Table 1). Evaluation of the anti-SARS-CoV-2 effect associated with UV-4B monotherapy regimens against the delta, and omicron variants of SARS-CoV-2 demonstrated that these strains were also susceptible to drug effect, yielding EC_50_ values of 1.60 and 0.507 μg/mL, respectively (Table 1). The results of monotherapy studies conducted with EIDD-1931 revealed that low μg/mL drug concentrations effectively inhibited the replication of all three variants. EIDD-1931 achieved EC_50_ values equal to 0.217 μg/mL against the beta variant, 0.506 μg/mL against the delta variant, and 0.080 μg/mL against the omicron variant strain (Table 1).

### 3.2. Combination Regimens of UV-4B and EIDD-1931 Enhance Antiviral Activity against the Beta, Delta, and Omicron BA.2 Variants of SARS-CoV-2

**Figure 1:**
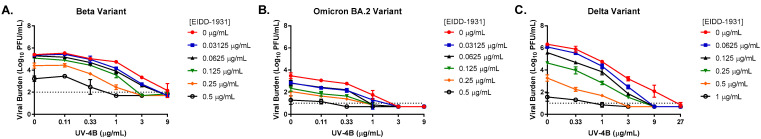
Antiviral activity of UV-4B and EIDD-1931 combination regimens against SARS-CoV-2. ACE2-A549 cells were infected with the (**A**) beta, (**B**) omicron, or (**C**) delta variants of SARS-CoV-2 at multiplicities of infection (MOIs) of 0.0075, 0.05, or 0.01, respectively. UV-4B, EIDD-1931, or both were added to cells following infection. Viral supernatant samples were collected on day 3 when peak viral titers were obtained in the no-treatment control arm. Levels of infectious virus were measured by plaque assay on Vero E6 or Vero E6-TMPRSS2-T2A-ACE2 cells and reported as log_10_ plaque forming units per milliliter (PFU/mL). Each data point corresponds to the mean of two independent samples; error bars represent one standard deviation. The dashed line corresponds to the assay limit of detection (100 PFU/mL for the beta variant and 10 PFU/mL for the omicron and delta variants).

Six concentrations of UV-4B ranging from 0 to 9 μg/mL and EIDD-1931 ranging from 0 to 0.5 μg/mL were evaluated both as mono- and combination therapy against the beta and omicron BA.2 variants of SARS-CoV-2 in ACE2-A549 cells (Figure 1A,B). Monotherapy studies with the delta variant revealed that higher concentrations of UV-4B and EIDD-1931 were required to completely suppress viral replication relative to those needed to curb replication of the beta and omicron BA.2 variants. Thus, drug concentrations ranging from 0 to 27 μg/mL (UV-4B) and 0–1 μg/mL (EIDD-1931) were evaluated against the delta variant strain of SARS-CoV-2 (Figure 1C).

In the absence of therapy, the beta variant achieved peak titers of 5.4 log_10_ PFU/mL 3 days after infection (Figure 1A). When utilized as monotherapy against this strain, UV-4B caused a robust antiviral effect as the highest evaluated concentrations, 3 and 9 μg/mL, caused infectious titers to fall by 2.1 and 3.3 log_10_ PFU/mL, respectively. EIDD-1931 monotherapy also caused considerable viral suppression since drug exposure yielded reductions in peak titers of up to 2.2 log_10_ PFU/mL. Combination regimens enhanced the antiviral effect relative to monotherapy. For example, the addition of 0.5 μg/mL EIDD-1931 to 0.33 μg/mL UV-4B caused a 2.9 log_10_ PFU/mL decrease in peak infectious titers when compared to the no-treatment control. This combination inhibited SARS-CoV-2 by an additional 2.6 log_10_ PFU/mL relative to 0.33 μg/mL UV-4B monotherapy and 0.7 log_10_ PFU/mL compared to 0.5 μg/mL EIDD-1931 alone. Further evidence of the enhanced activity associated with combination therapy was observed when 0.5 μg/mL EIDD-1931 was added to UV-4B concentrations ≥1 μg/mL, as these combinations completely curbed the production of infectious virus (Figure 1A).

Replication of the omicron BA.2 variant of SARS-CoV-2 was less robust compared to the beta variant since infection with this strain resulted in peak viral titers of approximately 3.5 log_10_ PFU/mL in the no-treatment control arm (Figure 1B). Single-agent therapy with UV-4B yielded reductions in viral titers ranging from 0.4 log_10_ PFU/mL to 2.8 log_10_ PFU/mL, while EIDD-1931 monotherapy caused levels of infectious virus to decline by up to 2.2 log_10_ PFU/mL. Similar to results against the beta variant, the addition of UV-4B to EIDD-1931 also augmented the antiviral effect against the omicron BA.2 strain, especially at UV-4B concentrations ≥1 μg/mL since the addition of 1 μg/mL UV-4B to all evaluated concentrations of EIDD-1931 caused viral titers to either approach or fall below the assay limit of detection (Figure 1B).

The delta variant reached peak titers of 6.3 log_10_ PFU/mL on day 3 (Figure 1C). Our results demonstrated that single-agent therapy with either UV-4B or EIDD-1931 inhibited replication of the delta variant in a concentration-dependent manner; however, we found that the drug effect was substantially enhanced when these agents were utilized as combination therapy. For example, treatment with 0.25 μg/mL EIDD-1931 + 1 μg/mL UV-4B inhibited viral replication by approximately 3.5 log_10_ PFU/mL relative to the no treatment control, while monotherapy at these concentrations suppressed titers by 1.7 and 1.6 log_10_ PFU/mL, respectively.

The Greco Universal Response Surface Approach (URSA) model was fit to the combination therapy data to characterize the drug–drug interaction for antiviral activity between UV-4B and EIDD-1931 against each variant. The model fit data sets for each variant well, yielding r^2^ values of 0.936 for the beta variant, 0.941 for the omicron variant, and 0.983 for the delta variant (Table 2). The estimates for the drug interaction parameter, α, were positive for these combination regimens against the beta (α = 18.08) and omicron variants (α = 25.54), indicating that the interaction between these compounds was not antagonistic. Although most of the 95% confidence intervals were positive, the lower bounds crossed zero for both the beta (95% CI: −2.705–38.86) and omicron variants (95% CI: −12.13–63.21), meaning that the interaction between UV-4B and EIDD-1931 against these strains is considered additive with a propensity toward synergy. Lastly, our results demonstrated that UV-4B and EIDD-1931 combinations inhibited the delta variant of SARS-CoV-2 in a synergistic fashion (α = 2.576, 95% CI: 0.919–4.233) (Table 2).

**Figure 2:**
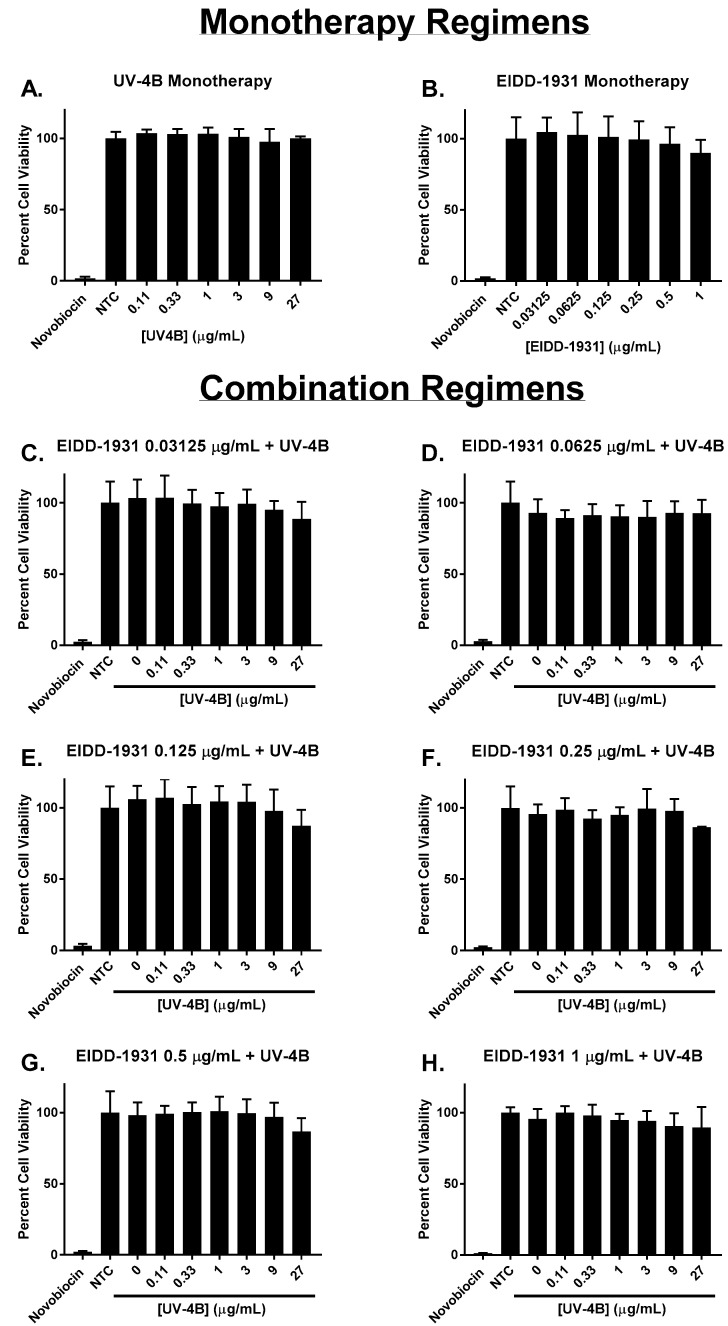
Viability of ACE2-A549 cells in the presence of UV-4B and/or EIDD-1931. Uninfected ACE2-A549 cells were treated with different concentrations of UV-4B, EIDD-1931, or both, and then cell viability was measured three days after treatment using the WST-1 cell proliferation assay. Percent cell viability was calculated relative to untreated cells (control arm). A 1000 μg/mL concentration of novobiocin was used as a positive control for cytotoxicity. Columns represent the mean of at least 6 replicates, and error bars correspond to one standard deviation.

Each drug’s effect on cell health and metabolism was measured via the WST-1 cell proliferation assay. The results of these studies demonstrated that UV-4B and EIDD-1931 as monotherapy were not toxic to ACE2-A549 cells at any of the concentrations evaluated in this work (Figure 2A,B). In addition, our findings indicated that cytotoxicity did not contribute to the antiviral activity associated with combinations of UV-4B plus EIDD-1931 since the calculated mean percent viability values of these regimens did not substantially differ from the control arm, and cell viability did not drop below 86% for all evaluated combinations (Figure 2C–H).

## 4. Discussion

Since its emergence in late 2019, SARS-CoV-2 quickly became a considerable threat to global public health due to its high transmissibility and substantial impacts on patient quality of life. Although great strides have been made to contain the pandemic through vaccination efforts, it remains imperative to develop effective antiviral regimens against SARS-CoV-2. The iminosugar antiviral UV-4B and the nucleoside polymerase inhibitor MOL have shown therapeutic promise against SARS-CoV-2 as monotherapy [9,11,26,27]. In this work, we evaluated the antiviral potential of these two agents, both as mono- and combination therapy against the SARS-CoV-2 beta, delta, and omicron BA.2 variants in a human lung cell line.

Results from our initial monotherapy studies demonstrated that UV-4B and EIDD-1931 effectively suppressed all three viral variants, yielding EC_50_ values in the low- to sub-µg/mL range (Table 1). It is important to note that the effective concentrations reported here for both drugs are physiologically achievable in man (Table 1) [13,28], highlighting the potential clinical utility of these agents against SARS-CoV-2. These findings suggest that UV-4B and EIDD-1931 exhibited the greatest antiviral activity against the omicron variant since calculated EC_50_ values were lowest against this strain; however, variant-specific differences in viral replication kinetics may have contributed to this effect. Replication of the omicron variant was not very robust in ACE2-A549 cells (yielding peak viral titers ≤4 log_10_ PFU/mL; Figure 1B). We hypothesize that since viral burden was lower with this variant, less drug was required to drive infectious titers toward the assay limit of detection resulting in lower EC_50_ values compared to the more robustly replicating beta and delta variants (Figure 1). Conversely, the delta variant achieved the highest peak viral burden (>6 log_10_ PFU/mL) compared to beta and omicron, and also yielded the highest EC_50_ values for both UV-4B and EIDD-1931 (Table 1). These data illustrate the influence of viral replication kinetics/viral burden on the potency of an antiviral agent.

Because UV-4B and EIDD-1931 target distinct proteins affecting separate stages of the viral replication cycle (viral assembly/release by UV-4B [14,15] and RNA synthesis by EIDD-1931 [16,17]), we hypothesized that their use in combination might enhance antiviral activity. The results from our preclinical studies, as well as estimates from the Greco URSA models, demonstrate that this was indeed the case. Combination regimens of UV-4B plus EIDD-1931 caused greater viral inhibition relative to each agent as monotherapy. The interaction between both antivirals was identified as additive with a propensity towards synergy for the beta and omicron BA.2 variants and synergistic against the delta variant.

Although the alpha interaction parameter for drug effect against the beta and omicron strains was highly positive, the interactions could not be characterized as synergistic due to the wide confidence intervals associated with these results. Parameter estimates show that the model did not fit the data as well for these data sets (r^2^ beta variant: 0.936; r^2^ omicron variant: 0.941) compared to results against the delta variant (r^2^ delta variant: 0.983). Out of all three variants, the delta strain also replicated the most robustly. We hypothesize that the model fit this dataset better and was able to more definitively characterize the interaction between both drugs as a result of the wider dynamic range of viral concentrations (range: 5.3 log_10_ PFU/mL) observed in the delta dataset in comparison to those from studies with the beta (range: 3.4 log_10_ PFU/mL) and omicron strains (range: 2.5 log_10_ PFU/mL).

Others have shown that combinations of available antivirals for COVID-19, including remdesivir and MOL, remdesivir and nirmatrelvir, and MOL and nirmatrelvir, are also additive against SARS-CoV-2 in vitro using CALU-3 human lung cells [29]. However, it should be noted that for these studies, the prodrug form of MOL and remdesivir were utilized and not the primary circulating metabolites (EIDD-1931 for MOL and GS-441524 for remdesivir). It is possible that the drug–drug interactions for antiviral effect will change slightly if the metabolites were evaluated in combination. Regardless, these studies, in addition to our study here, highlight the benefit of combination therapy over single-agent therapy regarding viral suppression.

Combinations of UV-4B plus EIDD-1931 drove infectious titers below the limit of detection at several evaluated concentrations in these studies. A high viral load has been correlated with both increased disease severity and a greater risk of viral transmission [30,31,32,33]. We hypothesize that the additional suppression elicited by combination therapy will induce a faster reduction in viral burden relative to what can be achieved with monotherapy alone. This effect can contribute to both improvements in patient time to recovery, and potentially shorten the duration of patient infectiousness, thereby helping to limit viral transmission. Moreover, combination therapy was also found to improve outcomes in a non-human primate model for SARS-CoV-2 [34]. In the study conducted by Rosenke and colleagues, MOL in combination with the protease inhibitor nirmatrelvir against the delta variant blunted disease progression, as infected animals treated with both drugs exhibited less severe manifestations of disease when compared to untreated animals or those that received treatment with only one drug [34].

Antiviral therapy is likely to be most effective when started early in the course of infection [35,36], and delays in the initiation of therapy can severely limit or nullify drug effect [37]. We hypothesize that delays in treatment initiation allow the virus to reach titers that are too high to be effectively cleared by approved antiviral regimens. However, it is plausible that the increase in antiviral effect from UV-4B and EIDD-1931 combinations will extend the window of time where initiation of treatment yields clinical improvements; this is an active area of investigation in the lab. In addition, the enhanced effect from combinations may also be useful in treating infection among patients hospitalized with severe infection, as currently approved regimens show limited effectiveness in this population of patients [35,38,39].

The emergence and rapid spread of SARS-CoV-2 strains with varying susceptibility to vaccines and monoclonal antibody therapies [7] necessitates the development of treatment regimens that exhibit pan-SARS-CoV-2 activity. Elsewhere, we have demonstrated that UV-4B effectively suppressed both a wild-type strain and the beta variant of SARS-CoV-2, causing only slight variations in EC_50_ values between strains [9]. We hypothesized that other SARS-CoV-2 variants would remain sensitive to UV-4B’s antiviral activity since the formation of several viral structural proteins is dependent on the host cell glycosylation machinery [40,41,42]. We also reasoned that EIDD-1931 would effectively suppress different SARS-CoV-2 strains because the structural motifs that make up the active binding site of the viral RNA polymerase are conserved both between SARS-CoV-2 variants as well as between SARS-CoV-2 and other coronaviruses [11,12,43]. Our findings demonstrated that both drugs effectively blunted viral titers of three different SARS-CoV-2 variants when used as monotherapy and as part of combination therapy. These results provide a strong indication that the regimens evaluated in this work are likely to provide antiviral protection regardless of viral variant, including future variants that have not yet emerged.

Combination therapy with two antivirals exhibiting distinct mechanisms of action has also been utilized as an approach to counter one of the main challenges associated with antiviral therapy, the emergence of resistance [44,45]. This strategy has successfully been applied to treat HIV and hepatitis C virus (HCV) infections, where agents with a low genetic barrier to resistance, such as protease inhibitors, are paired with one or more drugs belonging to a different antiviral class (i.e., nucleoside reverse transcriptase inhibitors (NRTIs) in the case of regimens to treat HIV and NS5A inhibitors for the treatment of chronic HCV infection) in order to prevent the emergence of resistant variants and treatment failure [44,46,47]. Although UV-4B and EIDD-1931 are predicted to exhibit a high genetic barrier to resistance when used as monotherapy [14,18,48,49,50,51], their use in combination can serve to further safeguard against the emergence of drug resistance.

A potential limitation of this study is that antiviral evaluations were conducted using static concentrations of UV-4B and EIDD-1931. This does not accurately recapitulate the pharmacokinetic (PK) profile associated with the oral administration of either agent in humans. Future investigations will focus on evaluating the antiviral effect of combination regimens under dynamic concentrations in which PK profiles associated with the clinical dosage regimen of each agent will be assessed through the use of the hollow fiber infection model. Another potential limitation is that the antiviral effect of each regimen was evaluated in vitro rather than in vivo. The use of an animal model was not deemed suitable in this study for several reasons. First, we believed that evaluations of the EIDD-1931 antiviral effect would be most generalizable to humans if preclinical evaluations were carried out using human-derived tissues. Our previous work with nucleoside analogues has shown that antiviral activity for these agents is heavily influenced by the cell type selected for antiviral evaluations, specifically the tissue and species from which the cells are derived [52,53]. This specific activity is most likely due to differences in drug uptake and metabolism into the active triphosphate form, as phosphorylation kinetics of nucleoside polymerase inhibitors vary between cell types [52,53,54,55]. Second, in vitro evaluations of combination therapy are an ideal first step in both identifying and narrowing down to the most promising combinations and exposures of each drug in the combination prior to animal experimentation. Because we implemented a fully factorial experimental design, evaluating multiple concentrations of each drug both alone and in combination, we are able to identify the most optimal two-drug combinations to further evaluate in an animal model. This experimental protocol not only provides the greatest amount of information, but also allows for the reduction of animal use.

Our findings demonstrate that combination regimens of UV-4B and MOL (EIDD-1931) are effective inhibitors of SARS-CoV-2 replication. Moreover, the enhanced antiviral activity observed in these studies suggests that combination therapy may be a promising strategy to maximize the effectiveness of antiviral therapy and, consequently, increase the possibility of altering the course of the pandemic. Further evaluations with different combinations of antivirals are also warranted.

## Figures and Tables

**Table 1:** Fifty percent effective concentration (EC_50_) values for UV-4B and EIDD-1931 monotherapy over the course of three days against the beta, delta, and omicron BA.2 variants of SARS-CoV-2. Fifty percent cytotoxic concentration (CC_50_) values for UV-4B and EIDD-1931 monotherapy in ACE2-A549 cells.

	EC_50_ [95% Confidence Interval (C.I.)]		CC_50_	
SARS-CoV-2 Variant	UV-4B (μg/mL)	EIDD-1931 (μg/mL)	UV-4B (μg/mL)	EIDD-1931 (μg/mL)
Beta	0.876 [0.718–1.084]	0.217 [0.142–0.323]	>27	>1
Delta	1.600 [0.927–2.790]	0.506 [0.483–0.531]		
Omicron BA.2	0.507 [0.490–0.522]	0.080 [0.290–0.383]		

**Table 2:** Parameter estimates from Greco URSA model for UV-4B and EIDD-1931 combination regimens.

Variant	Parameter	Units	UV-4B+EIDD-1931
Beta	r^2 a^	-	0.936
	E_con_ ^b^	Log10 PFU/mL	5.575
	EC_50_, _D1_ ^c^	μg/mL	0.714
	EC_50, D2_ ^d^	μg/mL	0.067
	m_1_ ^e^	-	1.358
	m_2_ ^f^	-	0.745
	α ^g^ (95% C.I.)	-	18.08 (−2.705–38.86 ^h^)
Omicron BA.2	r^2 a^	-	0.941
	E_con_^b^	Log10 PFU/mL	3.131
	EC_50_, _D1_ ^c^	μg/mL	3.081
	EC_50, D2_ ^d^	μg/mL	2.385
	m_1_ ^e^	-	0.445
	m_2_ ^f^	-	0.28
	α ^g^ (95% C.I.)	-	25.54 (−12.13–63.21 ^h^)
Delta	r^2 a^	-	0.983
	E_con_^b^	Log10 PFU/mL	6.478
	EC_50_, _D1_ ^c^	μg/mL	3.105
	EC_50, D2_ ^d^	μg/mL	0.478
	m_1_ ^e^	-	0.947
	m_2_ ^f^	-	1.447
	α ^g^ (95% C.I.)	-	2.576 (0.919–4.233 ^h^)

^a^ r^2^ corresponds to the goodness of fit of the model to the data; ^b^ E_con_ corresponds to viral replication in the absence of drug; ^c^ EC_50, D1_ is the concentration of UV-4B that produces a half-maximal antiviral effect; ^d^ EC_50, D2_ is the concentration of EIDD-1931 that causes half maximal antiviral effect; ^e^ m_1_ is the Hill constant for UV-4B; ^f^ m_2_ is the Hill constant for EIDD-1931; ^g^ α is the interaction parameter; ^h^ 95% confidence interval.

## Data Availability

The data presented in this study are available on request from the corresponding author.
